# Diabetic Patient Adherence to Yearly Influenza Vaccination in Northern Greece

**DOI:** 10.7759/cureus.22250

**Published:** 2022-02-15

**Authors:** Dimitrios Pilalas, Stylianos Daios, Melina Kachrimanidou, Georgia Kaiafa, Soultana Avgeri, Eleftheria Ztriva, Anastasia Kontana, Stergiani Keramari, Eleni Karlafti, Ilias Kanellos, Christos Savopoulos

**Affiliations:** 1 First Propaedeutic Department of Internal Medicine, University General Hospital of Thessaloniki AHEPA, Thessaloniki, GRC; 2 First Propedeutic Department of Internal Medicine, University General Hospital of Thessaloniki AHEPA, Thessaloniki, GRC; 3 First Department of Microbiology, University General Hospital of Thessaloniki AHEPA, Thessaloniki, GRC; 4 Bloodbank, Agios Pavlos Hospital, Thessaloniki, GRC; 5 Second Department of Paediatrics, University General Hospital of Thessaloniki AHEPA, Thessaloniki, GRC; 6 First Propedeutic Department of Internal Medicine, AHEPA University Hospital, Medical School, Aristotle University of Thessaloniki, Thessaloniki, GRC

**Keywords:** vaccination, diabetes, influenza, flu, adherence, risk factors

## Abstract

Background

Influenza virus infection is associated with increased morbidity and mortality in patients with diabetes mellitus. Public health authorities recommend yearly vaccination of diabetic patients against seasonal influenza.

Methods

We surveyed to define the adherence to influenza vaccination and associated factors among diabetic patients in Thessaloniki, Greece. Predictors of adherence to yearly influenza vaccination were assessed with logistic regression models.

Results

A total of 206 patients were enrolled, with 47.1% reporting yearly vaccination against influenza (95% confidence interval, CI:40.3% to 53.9%). In univariate models, the absence of additional indications for vaccination was associated with a decreased likelihood of vaccination uptake (OR:0.29, 95% CI:0.11 to 0.68, p=0.007); older diabetic patients were more likely to receive influenza vaccination (34% increase per 10 years of age). These associations were attenuated in multivariable analysis.

Conclusion

Our study demonstrates a significant gap in influenza vaccination coverage rate in diabetic patients. Our data could be extrapolated to enhance the uptake of vaccines against SARS-CoV-2: emphasis should be placed on patient education.

## Introduction

Seasonal influenza is caused by influenza A or B viruses affecting 5-15% of the population worldwide yearly [1]. Usually, seasonal influenza virus infections are associated with mild and self-limiting respiratory symptoms. However, complications may arise, resulting in increased morbidity and mortality. Host factors impact the severity of the disease: complications predominantly affect the elderly and patients with comorbidities such as diabetes mellitus (DM) [1]. Pro-coagulant effects caused by the influenza infection may exacerbate the increased risk of vascular disease associated with DM [1,2].

Impaired glucose metabolism is associated with dysfunction of innate and adaptive immunity. Public health authorities and scientific communities recommend the vaccination of patients with DM against seasonal influenza [2]. Nevertheless, the European Union (EU) Council 2009 goal to reach a vaccination coverage rate (VCR) of at least 75% across the European Union(EU) for at-risk populations has not been attained with only a few countries approaching the desired rate [3,4].

A high-level influenza VCR in the setting of the COVID-19 pandemic is a sine qua non to minimize the burden influenza would exert on hospitals. Diabetic patients are at high risk for severe complications and hospitalization, including increased mortality from SARS-CoV-2, and should be prioritized as a target population for vaccination [5,6]. Achieving optimal glucose control and taking all necessary precautions is of utmost importance to avoid excess morbidity and mortality and optimize healthcare resource allocation [6].

We aimed to estimate the influenza VCR among people with diabetes linked to medical care in our region and to identify factors associated with yearly seasonal influenza vaccination and missed opportunities.

## Materials and methods

A structured questionnaire was administered through telephone interviews conducted by one co-author (S.A) for four months. Patients eligible for the study were adults diagnosed with diabetes and followed in outpatient clinics of Agios Pavlos and the AHEPA University Hospital in Thessaloniki, Greece.

The questionnaire included demographic information(gender, age, marital status, number of children, if any, level of education, insurance status, and residence in urban, semi-urban, or rural areas) and diabetes care and patient comorbidities. Further questions addressed body mass index, smoking status, alcohol consumption. The respondents were asked if they were vaccinated yearly against seasonal influenza and to identify who proposed the vaccination during the study season. The final set of questions concerned aspects of diabetes care such as having the same doctor for the treatment of diabetes, the duration of the patient-doctor relationship, the number of visits per year, and the last HbA1c measurement. The documents required by the European and National Bioethics Committee were signed by the scientific council and the responsible departments.

Binomial 95% confidence intervals were calculated for proportions. Predictors of yearly vaccination against influenza were evaluated in univariate logistic regression models; those significantly associated with yearly vaccination (p<0.05) were tested in a multivariable logistic regression model by forced entry. All analyses were conducted in R version 4.0.0(packages binom and stats) [7].

## Results

A total of 206 adult diabetic patients were included. The mean age of the patient population was 67.7 years, and the male to female ratio was 1:1.4. All patients were insured. More than half of the patients lived in urban areas (65%) and had not received secondary education (62.6%). The demographic characteristics of the patient population are summarised in Table 1. Among survey participants, 83.3% had at least one child. The median body mass index was 29 (interquartile range, IQR: 25.7-33.2). Most patients were non-smokers (78.6%) and refused regular alcohol intake(95.6%). Approximately 86% of patients were diagnosed with DM Type II, and only one out of three had been diagnosed with diabetes for less than 10 years. Most patients had long-lasting patient-doctor relationships (median eight years, IQR 4-15) and visited their providers frequently (median four encounters/year, IQR: 2-5). However, only 33.5% had on target HbA1c levels.

**Table 1:** Demographic characteristics of diabetic patients linked to healthcare enrolled in a survey of adherence to yearly vaccination against influenza in Thessaloniki, Greece.

Patients enrolled	206
Mean age (standard deviation)	67.7 (13.6)
Gender (%)	
Male	87 (42.2%)
Female	119 (57.8)
Level of education in years (%)	
Less than 6	129 (62.7%)
7 to 9	21 (10.2%)
10 to 12	32 (15.5%)
More than 12	24 (11.6%)
Place of residence	
Urban	134 (65%)
Other	72 (35%)
Marital Status	
Married	149 (72.3%)
Other	56 (27.2%)
Unknown	1 (0.5%)

The majority of the patients had at least one other indication for flu vaccination except for diabetes (85.4%). Comorbidities included cardiac disease(38.8%), pulmonary disease (12.1%), malignancy (6.3%), immunosuppression (20.3%), chronic renal disease (18.4%), neurological disease (5.3%) and morbid obesity (3.9%).

In our study, the VCR against influenza yearly was 47.1% (97/206,95% confidence interval, CI: 40.3 - 53.9%). Univariate and multivariable logistic regression analysis results are reported in Table 2.

**Table 2:** Predictors of yearly adherence to influenza vaccination against influenza among diabetic patients linked to healthcare, Thessaloniki, Greece

Predictor	Univariate analysis		Multivariable analysis	
	Odds ratio (95% confidence interval)	p-value	Odds ratio (95% confidence interval)	p-value
Age (per 10-year increase)	1.34 (1.10 to 1.79)	0.006	1.22 (0.90 to 1.63)	0.15
Gender (reference male)	1.17 (0.67 to 2.04)	0.579	-	-
Level of education (reference education of less than 6 years)	0.59 (0.33 to 1.04)	0.072	-	-
Place of residence (reference urban)	1.20 (0.67 to 2.13)	0.539	-	-
Absence other than diabetes indication for flu vaccination (reference presence of any other indication)	0.29 (0.11 to 0.68)	0.007	0.46 (0.15 to 1.31)	0.158

It should be noted that among the patients who were vaccinated yearly, 53.6% reported being self-motivated to seek a vaccine prescription, 14.4% had the vaccination recommended by their primary care physician, and 26.8% by a specialty doctor. Notably, 5.2% of patients were vaccinated following a recommendation by their pharmacist.

## Discussion

We surveyed diabetic patients attending outpatient clinics in one tertiary and one secondary care hospital in Thessaloniki, Greece. The yearly influenza VCR was 47.1% (95% CI: 40.3% to 53.9%). Older age and other indications for flu vaccination were associated with an increased likelihood of vaccination against the flu in univariate analysis, but the association was attenuated in multivariable analysis. Almost half of the patients who reported yearly vaccination were self-conscious about the need to be vaccinated. In our sample, specialty doctors motivated the vaccination almost two times more frequently than primary care physicians(26.8% vs 14.4%).

Data concerning influenza VCR in populations at risk in Greece are limited, and the preponderance of the literature concerns healthcare workers [8-10]. Available influenza VCR data from the Survey of Health, Ageing and Retirement in Europe(SHARE) program collected in 2004-05 indicate that Greece had VCR lower than 27% in all high-risk groups [11]. In this survey, only 21.8%(95% CI:15.1% to 28.5%) of patients with diabetes were vaccinated against influenza with an OR of 1.07 (95% CI: 0.7 to 1.66) compared to non-diabetic individuals [10]. In a recent nationwide study in adults older than 60, 83.6% (95% CI 80.9% to 86.4%) of patients with diabetes reported being vaccinated against the flu versus 52.9% of diabetics over 60 reporting yearly vaccination in our study [12]. In the study by Papagiannis et al. [12], the sample of general practitioners was random, nationwide, geographically stratified and the patients enrolled subsequently were a convenience sample. In contrast, our study was based on a convenience sample from two hospitals. Nevertheless, it should be noted that the development of the primary care network in Greece does not cover the entire population at present, especially in urban centers, as reflected by the low percentage of primary care physicians motivating the vaccination in our study.

According to a report from the European Centre for Disease Prevention and Control in 2016-17, the influenza VCR among patients with chronic medical conditions ranged from 15.7% to 57.1%, with data available for only 7 of the 30 responding states [3]. Individual studies also reported VCRs below the 75% threshold in patients with diabetes [13-16].

The finding that older age increases the likelihood of vaccination against the flu is consistent with the literature [14,17-19], but in Europe, VCRs seem to be decreasing in the elderly [16]. Also alarming is the finding that people with diabetes without other comorbidities seem to neglect the need for vaccination against the flu; previous research in Greece supports our findings [20]. It should be noted that the associations mentioned above were attenuated in multivariable analysis.

Limitations of our study include the sample's representativeness, which may reflect a more complex patient population than the general diabetic population, and recall bias.

## Conclusions

In conclusion, our study demonstrates a significant gap in influenza VCR in diabetic patients despite official recommendations supporting the vaccination and the fact that vaccination is provided free of charge. Our data could be extrapolated to enhance the uptake of vaccines against SARS-CoV-2: emphasis should be placed on patient education.
